# Items, Selections and Remarks

**Published:** 1870-09

**Authors:** W. W. Miner


### Items, Selections and Remarks.

BY W. W. MINER, A. B.

Dr. G. C. S. Choate has resigned his position as Superintendent of the State Lunatic Asylum, at Taunton, Mass., and Dr W. W. Gooding, of the U. S. Hospital for the Insane, at Washington, has been appointed to the same.——Dr. Alexander Russell Simpson, nephew of the late Sir James Y. Simpson, has been appointed his successor in the chair of Obstetrics at the University of Edinburgh.——Rokitansky has recently been elected Corresponding Member of the Academy of Sciences of Paris. The names of Lebert and of Donders were also considered at the election.——Dr. Luther Parks has retired from the editorship of the Boston Medical and Surgical Journal, and Dr. Francis H. Brown is his successor.——Prof. Hamilton has resigned the chair of Surgery at Long Island Medical College, and Prof. Alpheus B. Crosby, of the University of Michigan is appointed to that position.——Prof. W. T. Lusk, of Long Island Medical College, has been appointed to deliver the course of lectures on Physiology at Harvard Medical College, the ensuing Winter.——Sir William Ferguson, Bart., has been elected President of the Royal College of Surgeons, London. ——The New York State Hospital for Diseases of the Nervous System, situated at the corner of second Avenue and St. Mark's Place, New York City, was opened to the public in July. Drs. Hammond, Vance, Flint, Elliott, Wood, Sayre, and Cross, constitute its Board of Medical Officers. It is desired that this institution shall be entirely a gratuitous one and connected with it, is to be an out-door service in which patients are treated at their homes. No incurable cases are to be admitted to the Hospital. The Trustees, in a circular addressed to the public, state that the cost of the yearly support of one bed is estimated at $350, and contributions of permanent funds by individuals, are earnestly desired.——A Pathological Laboratory has been opened at Bellevue Hospital where students may receive a thorough course of instruction in Pathological Anatomy and Histology. The instructors are Drs. Janeway, Delafield, Lusk, and Buck.——A call has been issued by the regular practitioners of California for a meeting at San Francisco, October 16th, for the purpose of re-organizing the State Medical Society.——A Hospital for Diseases of Women is to be erected in honor of the memory of the late Sir James Y. Simpson; and it is to be planned in accordance with the views Prof. Simpson lately expressed respecting the arrangement of a hospital system.——A work entitled "Ovarian Tumors, with special reference to Ovariotomy," by Prof. E. R. Peaslee, M. D., is soon to be published by Appleton & Co.——The fourth German edition of Prof. Billroth's Lectures on General Surgical Pathology, has been translated by Chas. E. Hackley, M. D., of New York, and is about to be issued from the press of D. Appleton & Co.—Prof Wm. A. Hammond has written a work on "Diseases of the Nervous System, which is now in press.——A work on Epilepsy, by Prof. Echeverria, of New York, is soon to appear.——Dr. J. C. Dalton's work on Physiology and Hygiene, has been translated into French.——Dr. N. S. Davis, in his address before the Medical Journal Association, states that of the thirteen or more medical periodicals now being published in the United States, only thirteen have been published more than a single decade. ——Dr. Loebel, of Vienna, in a patient suffering from dyspnœa in pericarditis tapped the pericardium and removed about three ounces serum, which afforded great temporary relief. Hydrothorax soon supervened and on the twelfth day he made a second puncture and removed an equal amount of fluid. The patient, sixty-eight years of age, died the next day and hydrothorax was found on both sides, but the pericardium contained very little fluid and no traces of blood or lesion of the heart itself could be found.——The well-known gynecologist, Dr. Gunning S. Bedford, died in the early part of the present month at his residence in New York City.——Dr. Syme, of Edinburg and one of the colleagues of the late Sir James Y. Simpson, has recently died.—Sir James Clarke, physician to the Queen, died in London, June 20th, at the age of eighty-two——The death of Prof. Albrecht Von Graefe, the distinguished ophthalmologist and one of the brightest stars of the profession, occured at Berlin in the latter part of July.——James Copeland, M. D., F. R. S., widely known as the author of the "Encyclopædic Dictionary of the Medical Sciences," died at London, July 12th.——German papers say that in New York, in the case of a criminal who appeared to be insane, chloroform was given and the feigned character of the insanity became apparent.——Prof. N. S. Davis, of Chicago Medical College, announces that female matriculants will no longer be received at that institution, since that, "although the ladies were well treated by the young men, and no serious difficulty occurred, yet the patients objected to appearing in the clinic before mixed classes.——The Royal College of Surgeons, London, has decided to recognize certificates of medical education from the schools of medicine in New York, Boston and Philadelphia.

